# Optimization and Testing of a Commercial Viability PCR Protocol to Detect *Escherichia coli* in Whole Blood

**DOI:** 10.3390/microorganisms12040765

**Published:** 2024-04-10

**Authors:** Kristi L. Jones, Federico Cunha, Segundo Casaro, Klibs N. Galvão

**Affiliations:** Department of Large Animal Clinical Sciences, University of Florida College of Veterinary Medicine, Gainesville, FL 32608, USA

**Keywords:** viability PCR (vPCR), bacteremia, method comparison, blood, propidium monoazide (PMA)

## Abstract

Bacteremia, specifically if progressed to sepsis, poses a time-sensitive threat to human and animal health. *Escherichia coli* is a main causative agent of sepsis in humans. The objective was to evaluate a propidium monoazide (PMA)-based viability PCR (vPCR) protocol to detect and quantify live *E. coli* from whole blood. We optimized the protocol by adding a eukaryotic-specific lysis step prior to PMA exposure, then used spiking experiments to determine the lower limit of detection (LOD) and linear range of quantification. We also compared the vPCR quantification method to standard colony count of spiked inoculum. Lastly, we calculated percent viability in spiked samples containing 50% live cells or 0% live cells. The LOD was 10^2^ CFU/mL for samples containing live cells only and samples with mixed live and heat-killed cells. The linear range of quantification was 10^2^ CFU/mL to 10^8^ CFU/mL (R^2^ of 0.997) in samples containing only live cells and 10^3^ CFU/mL to 10^8^ CFU/mL (R^2^ of 0.998) in samples containing live plus heat-killed cells. A Bland–Altman analysis showed that vPCR quantification overestimates compared to standard plate count of the spiked inoculum, with an average bias of 1.85 Log_10_ CFU/mL across the linear range when only live cells were present in the sample and 1.98 Log_10_ CFU/mL when live plus heat-killed cells were present. Lastly, percent viability calculations showed an average 89.5% viable cells for samples containing 50% live cells and an average 19.3% for samples containing 0% live cells. In summary, this optimized protocol can detect and quantify viable *E. coli* in blood in the presence of heat-killed cells. Additionally, the data presented here provide the groundwork for further development of vPCR to detect and quantify live bacteria in blood in clinical settings.

## 1. Introduction

Bacteremia is the presence of viable bacteria in the blood and is of clinical relevance when it elicits a systemic inflammatory response and subsequent sepsis. Although both Gram-negative and -positive bacteria can cause sepsis, Gram-negative bacteria are reported to have about 10% higher prevalence than Gram-positive bacteria. *E. coli* is the main Gram-negative causative agent [1,2,3]. The current standard for detection of viable bacteria in blood is automated culture systems [4]. These methods use specialized culture media and colorimetric or fluorescent detection systems for continuous monitoring of metabolic by-products resulting from replicating bacteria [4]. The average time for detection of 1.9 to 42 CFU/mL of *E. coli* seeded into blood using automated systems has been reported to be 9 to 12 h, depending on the system [5,6]. Despite this low detection limit, 40–50% of patients treated for sepsis are culture-negative [7,8]. Furthermore, clinical signs and mortality rates were the same for culture-positive and -negative patients diagnosed with sepsis [9]. This indicates a high frequency of false-negative results in the automated blood culture gold standard method.

Common molecular methodologies such as mass spectrometry proteotyping, qPCR, and FISH have been investigated as alternatives to culture-based detection of bacteremia [10,11,12,13]. Despite these alternative detection methods, none of these molecular approaches provide information on viability status of detected microbes.

As the name implies, viability PCR (vPCR) is an alternative to culture-based methods capable of identifying live cells. Briefly, this method consists of exposure to DNA binding dye followed by quantitative PCR [14]. Propidium monoazide (PMA) is the most common viability stain used. Upon photoactivation, PMA covalently binds to exposed DNA and prevents subsequent amplification of the target gene during qPCR [15]. PMA is unable to penetrate the intact cell membrane of live bacterial cells and, thus, will not covalently bind DNA. Hence, quantification of gene targets following PMA exposure are representative of live bacteria in the sample. This method has been proposed for use in food safety and clinical settings to detect both Gram-negative and -positive pathogenic bacteria [16,17,18,19,20]. 

The objective of our study was to evaluate a PMA-based vPCR protocol to detect and quantify live *E. coli* from whole blood. Herein we report optimization of the protocol by adding a eukaryotic-specific lysis step prior to PMA exposure. We used spiking experiments to determine the lower limit of detection and linear range of quantification. We also compared the vPCR quantification method to standard colony count of spiked inoculum. Lastly, we calculated percent viability in spiked samples containing 50% live cells or 0% live cells, both in the presence of heat-killed cells. This is the first report of use of vPCR to detect and quantify *E. coli* from blood. 

## 2. Materials and Methods

### 2.1. Method Overview

**Figure 1:**
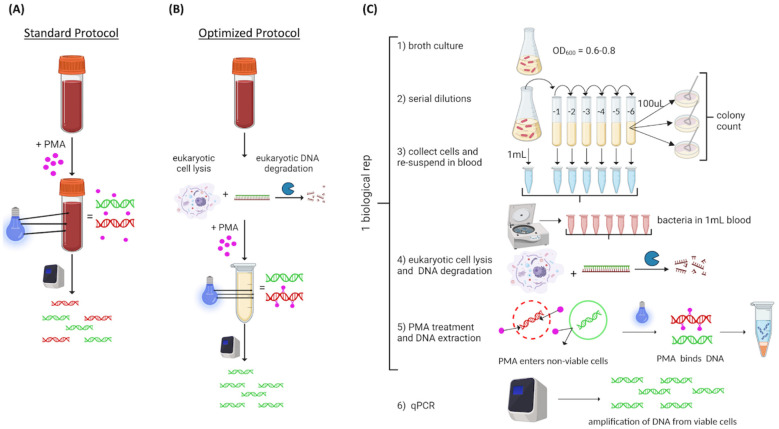
Illustration of experimental overview: red alpha helix represents DNA from heat-killed cells, and green alpha helix represents DNA from live cells. Optimization experiment: experiment was conducted independently for live and heat-killed cells using standard and optimized protocols. (**A**) Standard protocol. (**B**) Optimized protocol. Testing experiments were conducted using live cells only or live plus heat-killed cells as indicated. Steps 1 to 5 represent 1 biological replicate (**C**). Illustration created using Biorender (Biorender.com).

We used a commercially available PMA-based vPCR protocol (PMA Real-Time PCR Bacterial Viability Kit—E. coli (uidA), Biotium, Inc., Freemont, CA, USA, catalog number 31050-X). The experimental approach is illustrated in Figure 1. Commercial blood was spiked with 10^8^ live or heat-killed *E. coli*, then exposed to PMA (PMAxx^TM^, Biotium Inc., Freemont, CA, USA) using the standard protocol described by the manufacturer (Figure 1A). We hypothesized that the red color of the blood could be a limiting factor for PMA binding efficiency; therefore, we added a eukaryotic cell lysis step prior to PMA exposure to optimize the protocol (Figure 1B). Following optimization, the experimental design consisted of *E. coli* grown to an OD_600_ of 0.6 to 0.8 that was serially diluted ten-fold from 10^8^ to 10^2^ CFU/mL; bacterial concentration was confirmed by standard plate counts. Bacterial cells from 1 mL of each dilution were collected by centrifugation and resuspended in 1 mL commercial blood. For samples containing heat-killed cells, 1 mL aliquots of 10^7^ CFU/mL were heat-killed at 95 °C, collected, resuspended, then added to the corresponding blood sample. Samples were treated with eukaryotic lysis buffer. PMA was added and activated by light (PMAlite^TM^, Biotium Inc.). DNA was extracted and qPCR was conducted (Figure 1C). Unless otherwise stated, we used 5 biological replicates consistent with recommendations of Ma et al. [21].

### 2.2. Bacteria Strain and Preparation for Spiking 

We used *E. coli* strain KG-15 isolated from cow feces. The frozen glycerol stock was revived and maintained on CHROMagar™ *E. coli* (CHROMagar™). For each experiment, a single colony was inoculated into 10 mL brain hear infusion (BHI) broth (Remel) and incubated overnight at 37 °C. The culture was back-diluted to OD_600_ = 0.1 in BHI broth and grown to OD_600_ = 0.6–0.8 for all experiments. A series of 10-fold dilutions of live bacteria was prepared in BHI broth. For each dilution, cells from 1 mL were collected by centrifugation, supernatant was discarded, and cells were resuspended in 1 mL commercial blood (sheep blood in citrate, Hemostat). Standard plate counts on BHI agar were conducted in triplicate; briefly, 100 µL of the 10^−6^ dilution was plated onto BHI, incubated, and colonies were counted the following day. 

For heat killing, a 1 mL aliquot of 10^7^ CFU/mL dilution was incubated at 95 °C for 20 min, and cells were collected by centrifugation. The pellet was resuspended in 1 mL blood for samples containing only heat-killed cells. For samples containing live plus heat-killed, the pellet was resuspended in 50 µL BHI and this total volume was transferred to the appropriate blood sample containing the live cells. Effective heat killing using this method was verified by standard plate count of 0 CFU/mL on BHI agar in triplicate for each experiment.

### 2.3. Optimization of PMA Exposure in Whole Blood 

To determine if inclusion of a eukaryotic lysis step would improve the overall vPCR results, whole blood was spiked with 10^8^ live or heat-killed *E. coli* cells and then processed following the standard protocol or with the eukaryotic lysis step prior to PMA exposure (Figure 1A). The ΔCt between live and heat-killed cells was compared. The optimized protocol included a eukaryotic specific lysis step and subsequent DNA depletion step adapted from the Zymo HostZERO microbial DNA kit (Zymo Research Corporation, Irvine, CA, USA). Specifically, we mixed 1 mL spiked blood with 3 mL commercial red blood cell lysis solution (Zymo, catalog number R1022-2-100) at room temperature for 15 min. Then, cells were collected by centrifugation and resuspended in 200 µL PBS, and 1 mL Host DNA Depletion Solution (Zymo, catalog number D4310-1-20) was added. Samples were incubated at room temperature for 15 min, and bacteria cells were collected by centrifugation. Pelleted bacterial cells were re-suspended in BHI for subsequent PMA exposure. 

For all experiments, sample exposure to PMA was performed as described in the manufacturer’s protocol using the enhancer and PMA solution at a final concentration of 25 µM [22]. PMA was made fresh for each experiment and protected from light. Samples were incubated at room temperature with rotation for 15 min and then exposed to light for 20 min using the PMAlite^TM^. All steps involving PMA were conducted in low light, and samples were covered during incubation steps until DNA extraction. Samples were placed on ice until DNA extraction for a maximum of 1 h. Efficiency of this exposure protocol was tested by calculating the ΔCt between samples with or without PMA exposure. This was done for both live and heat-killed cells spiked into whole blood as described for controls in the manufacturer’s protocol. The expected results are a ΔCt between +/− PMA less than 1 cycle for live cells and >4 cycles for heat-killed cells. A two-tailed t test was used to determine significance using 3 biological replicates.

### 2.4. DNA Extraction 

DNA was extracted per the manufacturer’s recommended protocol (Qiagen, Hilden, Germany, QIAamp DNA Mini kit catalog #51306). Briefly, samples were lysed with protease K and lysis buffer, ethanol was added to precipitate DNA, and then samples were transferred to a spin column. Samples were washed, and DNA was eluted in a 75 uL elution buffer provided by the manufacturer. DNA was stored at −20 °C until qPCR. 

### 2.5. Preparation of Standard Curve DNA 

*E. coli* cells were grown as described above. Cells from 1 mL of stock solution, OD_600_ = 0.6–0.8 equivalent to 10^8^ CFU/mL, were collected by centrifugation, supernatant was carefully discarded, and the pellet was resuspended in BHI. Samples were exposed to PMA and DNA extracted as described above. DNA concentration was measured using a Qubit broad range dsDNA kit (Thermo Fisher Q32850, Eugene, OR, USA). Tenfold serial dilutions, ranging from 15.5 ng to 15.5 fg, were made in elution buffer. The DNA concentration was plotted against the Ct values to determine linearity of the standards automatically in the QuantStudio™ Design & Analysis Software v1.5.2. 

### 2.6. Real-Time Quantitative PCR 

Commercially available master mix and *uidA* primer mix (Biotium, catalog number 31050-X) were used. Rox was used as a reference dye in accordance with thermocycler recommendations. Each reaction contained 2uL of DNA template and was run in triplicate on StudioQuant3 thermocycler (Thermo Fisher). Cycling parameters are as described in the product insert, and melt curve analysis was included. 

Copy number per sample was calculated using the standard formula (# copies = (g DNA from standard curve × 6.0221 × 10^23^ molecules/mole)/(length of amplicon × 660 g/mole)). BLAST (NCBI.gov) was used to verify presence and copy number of *uidA*, in KG-15. The gene sequence was obtained from EcoCyc (accession ID EG11055), and the KG-15 whole genome sequence (accession number PVOG00000000.1) was obtained from NCBI.

### 2.7. Evaluation of Analytical Parameters

The lower limit of detection (LOD) was determined based on the lowest spiked *E. coli* concentration to produce a positive qPCR result. The qPCR result was positive if the average Ct of 3 technical replicates was 3 cycles greater than the Ct of the NTC [23]. 

Linear regression of Ct versus Log_10_ CFU/mL spike concentration and was determined using Excel [21]. Inter- and intra-assay variability was evaluated based on calculated copy number CV%. Ma et al. report an acceptable copy number CV% for inter-assay variability is 45% and intra-assay variability is 25% based on five plates [21]. Our experiments used 5 biological replicates to determine inter-assay variability across the whole protocol and triplicate technical replicates to determine intra-assay variability of the qPCR reaction. We used biological replicates to determine variability because the objective was to evaluate the PMA exposure, not just the qPCR parameters as we were using a commercial kit for amplification. 

### 2.8. Method Comparison

The Bland–Altman tool in Sigma-Plot (version 14.5) was used for the method comparison. Standard plate count was used to determine CFU/mL spiked into each sample as the reference method; vPCR was the test method. BLAST analysis confirmed *uidA* as a single copy gene in KG15. Therefore, we assumed a single copy calculated from qPCR is equivalent to a single CFU. Five data points for each concentration, corresponding to the five biological replicates, were used. The first graph plots the Log_10_ copy number against the CFU/mL colony count, calculates a regression line and 95% confidence interval, and plots a trueness line. This analysis provides a visual representation of how the test method compares to the reference method. The second analysis determines bias. This method plots the average of the two methods against the difference, and indicates the mean and upper and lower limits of agreement based on a 95% confidence interval. We used samples spiked with 10^3^ to 10^8^ *E. coli* for this analysis. 

### 2.9. Percent Viable Cells

Percent viable cells in whole blood spiked with either 10^7^ live and 10^7^ heat-killed CFU/mL or no live cells and 10^7^ heat-killed CFU/mL was calculated as suggested by the manufacturer with modifications. Specifically, duplicate samples were prepared for each spiking concentration; one sample was treated with PMA, and one was not. DNA was extracted and qPCR was conducted as described above. Percent viability was calculated using the formula provide modified as follows to account for PCR efficiency. We used PCR efficiency for targets without PMA treatment from preliminary data investigating this kit. PCR efficiency without PMA treatment was 111%, and PCR efficiency with PMA treatment was 92%. Fold effect was calculated as described previously to account for PCR efficiency [24] and then used in the percent viability calculation recommended by the manufacturer as described below. 

ΔCt was calculated:

ΔCt = Ct without PMA treatment − Ct with PMA treatment.

Percent viable cells in the population was calculated:

% viable = 100/(E_target_^ΔCt^/E_reference_^ΔCt^)

where E is PCR efficiency, target is with PMA treatment, and reference is without PMA treatment. 

## 3. Results

### 3.1. Optimization of PMA Exposure in Whole Blood 

**Figure 2:**
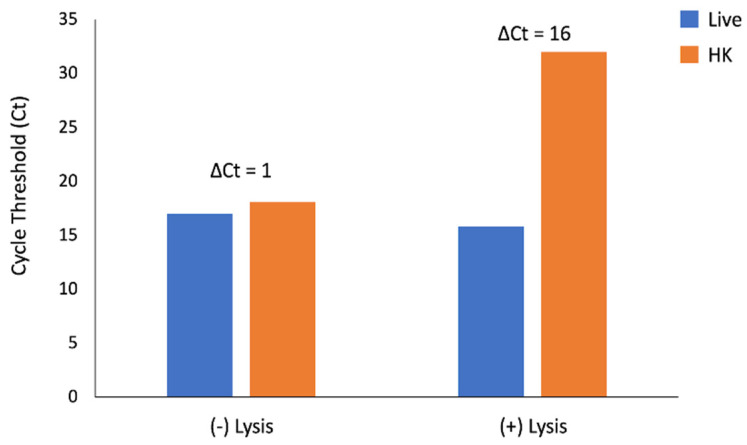
Cycle threshold (Ct) differences between live and heat-killed (HK) *E. coli* spiked into whole blood with and without eukaryotic lysis prior to PMA treatment. NTC Ct = 37.8. Results from one biological replicate.

The ΔCt for amplification of *uidA* between live and heat-killed samples without pre-treatment with eukaryotic cell lysis was 1 cycle. The addition of a eukaryotic cell lysis step prior to PMA exposure increased the ΔCt to 16 cycles (Figure 2). 

**Figure 3:**
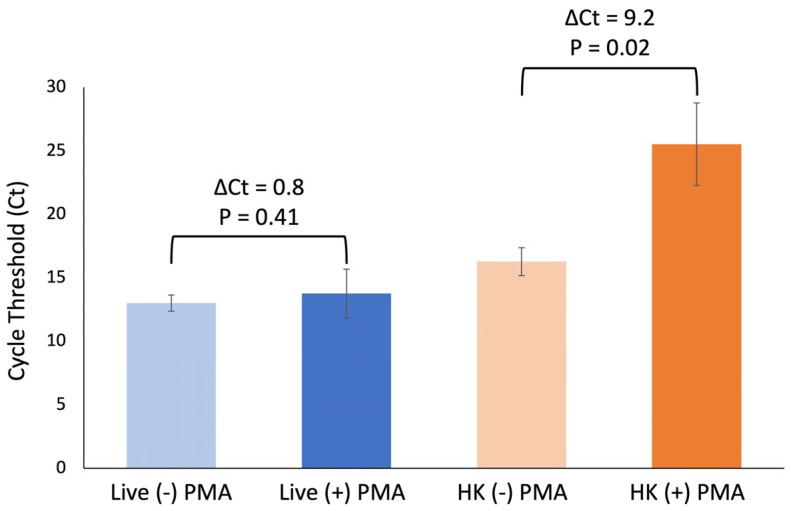
PMA inhibition of amplification when *E. coli* cells were heat-killed and no significant inhibition on amplification for live cells. Bars indicate standard error. Two-tailed paired t test was used to calculate *p* values reported here. Average of 3 biological replicates.

Next, we verified the PMA exposure using the conditions described here adequately inhibited amplification of DNA from heat-killed cells without impacting DNA amplification of live cells. Using three biological replicates, an average ΔCt of 9.2 cycles was observed for samples containing heat-killed cells only, while an average ΔCt of 0.8 was observed for samples containing live cells only (Figure 3). There is a significant difference between +/− PMA in samples containing heat-killed cells only: *p* = 0.02. However, no significant difference was observed for the same comparison in samples containing live cells only: *p* = 0.41. 

Taken together, these data indicate that this optimization step is necessary for differentiation of live and heat-killed *E. coli* and that these conditions do not adversely impact DNA amplification of live cells.

### 3.2. Evaluation of Analytical Parameters

The LOD was determined to be 10^2^ CFU/mL for both live only and live plus heat-killed spiked blood samples. When only live cells were spiked into blood, five out of five biological replicates produced a positive result in samples corresponding to 10^2^ CFU/mL spike concentrations. Although samples corresponding to 10^1^ CFU/mL were tested, zero out of three produced a positive result; thus, this concentration was eliminated from subsequent experiments. Likewise, blood spiked with live plus heat-killed *E. coli* produced a positive result in samples corresponding to 10^2^ CFU/mL spike concentrations in the presence of 10^7^ CFU/mL heat-killed cells in five out of five biological replicates. 

**Figure 4:**
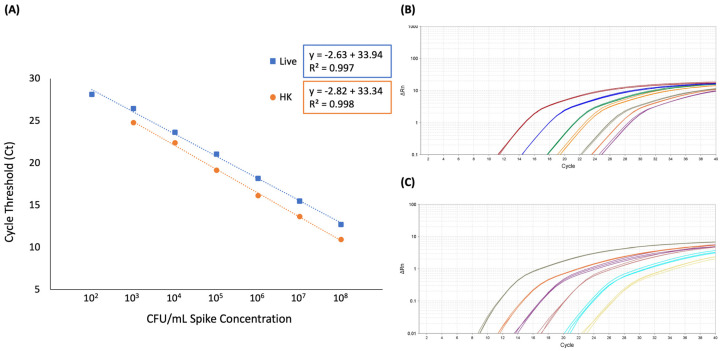
(**A**) Linear regression of spike CFU/mL and Ct value. Blue data points and dotted line represent average of 5 biological replicates of samples containing only live cells. Equation outlined in blue. Orange data points represent average of 5 biological samples containing live plus heat-killed cells. Equation outlined in orange. (**B**) Representative amplification plot of fluorescent signal of samples containing live cells only. (**C**) Representative amplification plot of fluorescent signal of samples containing live plus heat-killed cells.

The linear range of quantification for this method in whole blood was determined to be 10^2^ to 10^8^ CFU/mL with an R^2^ of 0.997 for samples containing live *E. coli* only and 10^3^ to 10^8^ CFU/mL with an R^2^ of 0.998 for samples containing live plus heat-killed *E. coli* (Figure 4 and Supplemental Table S1). 

Average calculated copy number inter-assay variability across the detectable range, was 6.2% and 4.1% for live cells only and live plus heat-killed cells, respectively (Supplemental Table S2). Both live cells only and live plus heat-killed cells showed greater variability at lower concentrations, as expected. Specifically, the range for samples containing live cells only was 4.6 to 10.1% and 1.3 to 9.5% for samples containing live plus heat-killed cells. All samples were below the recommended CV% cutoffs of 45% for inter-assay variability [21]. Average calculated copy number intra-assay variability across the detectable range was 7.2 and 9.6% for live cells only and live plus heat-killed cells, respectively (Supplemental Table S2). The range for samples containing live cells only was 3.6 to 10.0% and 3.1 to 16.2% for samples containing live plus heat-killed cells. Additionally, the Ct inter-assay average standard deviation over the detectable range for live cells only was 1.79 cycles, ranging from 1.25 to 2.44 cycles. The Ct inter-assay average standard deviation over the detectable range for live plus heat-killed cells was 1.11 cycles, ranging from 0.47 to 2.20 cycles. Intra-assay average standard deviation for Ct values of biological replicate one was 0.10 cycles with a range from 0.01 to 0.23 cycles over the detectable range for live cells only. Similarly, intra-assay average standard deviation for Ct values of biological replicate one was 0.14 cycles with a range from 0.06 to 0.24 cycles over the detectable range for live plus heat-killed cells (Supplemental Table S2). Taken together, these data indicate that the optimized vPCR method described here has a LOD of 10^2^ CFU/mL with a linear quantification range of 10^3^ to 10^8^ CFU/mL for live *E. coli* in the presence of heat-killed cells, and variability was within the acceptable ranges. 

### 3.3. Method Comparison 

**Figure 5:**
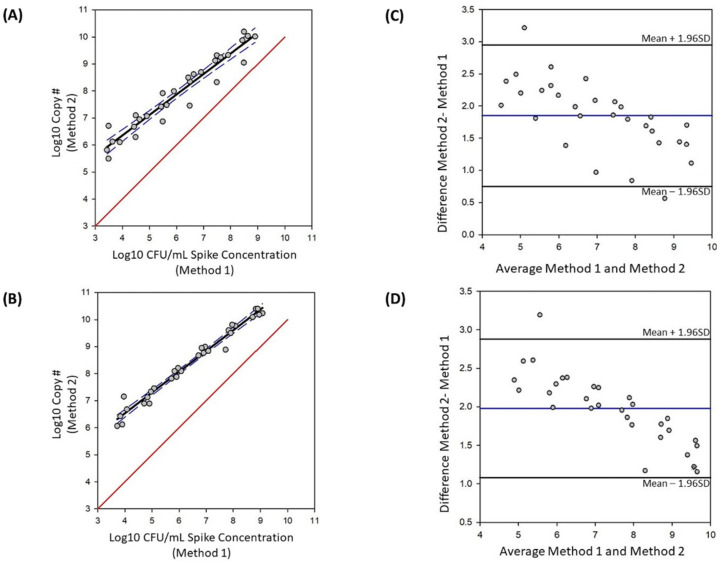
Method comparison between vPCR and spiked CFU/mL standard plate count quantification. Regression of Log_10_ CFU/mL compared to Log_10_ copy number for samples containing live cells only (**A**) and samples containing live plus heat-killed cells (**B**). Grey dots are sample points from 5 biological replicates at each concentration, red solid line is line of equality, black solid line is linear regression line, and blue dashed lines are 95% confidence intervals. Bland–Altman plots showing comparability and bias between quantification methods for samples containing live cells only (**C**) and live plus heat-killed (**D**). Blue line is the mean between the 2 methods for each concentration, and black solid lines are 1.96 SD +/− mean denoting the limits of agreement.

Our method comparison analysis showed an overestimate when vPCR is used for quantification compared to spiked colony counts, as indicated by all data points being above the line of equality in both live and live plus heat-killed samples (Figure 5A,B). Additionally, this analysis showed a proportional bias for both live and live plus heat- killed samples as indicated by closer agreement between the two methods at the higher spike concentrations (Figure 5). The calculated average bias was 1.85 Log_10_ CFU/mL for live only samples and 1.98 Log_10_ CFU/mL for live plus heat-killed samples (Figure 5C,D). The limits of agreement for samples with only live cells are 0.75 and 2.95 Log_10_ CFU/mL, and shifts to 1.09 and 2.88 Log_10_ CFU/mL for samples with live plus heat-killed cells (Figure 5). Taken together this data indicate that, compared to standard plate count, the optimized vPCR method described here overestimates live CFU/mL when absolute quantification is used. 

### 3.4. Percent Viable 

For samples containing equal concentrations of live and heat-killed *E. coli*, the average calculated percent viable from five biological replicates was 89.5%, ranging from 65.4 to 100.2%. The average ΔCt was 0.7 cycles for samples containing equal concentration live and heat-killed cells. Interestingly, four of the five replicates had differences in Ct for samples containing equal concentration live and heat-killed cells below the manufacture’s criteria of ≤1 cycle difference for live controls. Specifically, the calculated ΔCt was 2.3, 0.01, 0.05, 0.18, and 0.8 cycles for each of five biological replicates. 

For samples spiked with only heat-killed cells, the average calculated percent viable from five biological replicates was 19.3%, ranging from 12.3 to 29.8%. The average ΔCt was 9 cycles. All the ΔCt for the heat-killed samples were above the manufacture’s criteria of ≥4 cycles different for dead controls. Specifically, the ΔCt was 11.1, 10.2, 6.4, 7.7, and 9.8 cycles for each of the five replicates. Taken together, these data indicate that the relative quantity calculations result in an overestimation of live cells. 

## 4. Discussion

Herein we optimized a vPCR method to detect live *E. coli* in whole blood by the addition of a lysis step prior to PMA exposure. Previous reports utilizing vPCR and the manufacturer’s protocol use washes or relatively clear liquids. Due to the red color of whole blood, we anticipated inefficient penetration of light through the sample resulting in low covalent binding to exposed DNA. Since the manufacturer’s protocol states that the live cell control will have a ΔCT ≤ 1, we concluded that the observed 1 cycle difference between live and heat-killed cells would not consistently differentiate live and dead bacteria. However, with the addition of eukaryotic lysis prior to PMA exposure, the observed ΔCt was 16 cycles between live and heat-killed cells. Thus, we concluded that the addition of this optimization step allows for adequate differentiation between live and dead *E. coli*. Additionally, the optimized PMA exposure protocol described here performed as expected. The ΔCt for heat-killed cells was 9.2, greater than the manufacturer’s indicated 4 cycles for dead controls and below 1 cycle for live controls.

The LOD reported here is consistent with previous vPCR reports for *Vibrio parahaemolyticus* in shrimp and *E. coli* O157:H7 in ground beef [22,25]. Nonetheless, the LOD reported here is higher than the automated culture system [5,6]. The results reported here for linear range of quantification are consistent with previous vPCR reports [22,25,26]. Inter- and intra- assay variability results reported here are below previously suggested cutoffs and are within ranges previously reported for vPCR [21,26,27]. Although the analytical parameters of our optimized vPCR protocol are in line with previous reports of vPCR, further optimization is needed to lower the LOD. Furthermore, internal quality control standards similar to those proposed for *Campylobacter* [28] need to be developed to account for known variability in instrument and lab workflow that can limit quantitative PCR methodologies.

The objective of the method comparison analysis was to determine how accurately this vPCR method could quantify a known concentration of bacteria within the linear range in the presence of heat-killed cells. Indeed, we showed vPCR could quantify viable *E. coli* cells in the presence of heat-killed *E. coli*. However, this vPCR method overestimated viable cells in the presence of heat-killed cells with a bias of 1.98 Log_10_ CFU/mL. To determine if the heat-killed cells were the source of this overestimate, we conducted the method comparison analysis in samples containing only live cells. Again, quantification of viable cells from vPCR overestimated live cells with a bias of 1.85 Log_10_ CFU/mL. This observed overestimate indicates that the presence of heat-killed cells contributes to, but alone cannot explain, the observed overestimate. The source of the observed overestimate is not clear, but one possible explanation is that the vPCR quantification is detecting cells in a viable but non-culturable (VBNC) state that are not accounted for in the reference plate count method. Cells in the VBNC state are viable and thus have an intact cell wall; however, they are not readily culturable using standard culture methods. Since the vPCR method optimized here relies on an intact cell wall to prevent the PMA dye from interacting with DNA, cells in this state will be identified by this method as viable. However, since standard plate counts were used as a reference, these viable cells were not captured in this reference method, thus resulting in a higher number of viable cells being identified in vPCR compared to plate count. It has been reported that the VBNC subpopulation of cells ranges from less than 1% to 25% of the total populations of *E. coli* cells in stationary phase [29,30,31]. The possible presence of VBNC cells could contribute to, but does not explain, the observed overestimate. This observed overestimate using vPCR to quantify live *E. coli* is consistent with incomplete removal of target DNA, a known limitation of the vPCR method [32]. Although the protocol described here introduces a critical optimization step to mitigating this limitation, the PMA exposure parameters could be fine-tuned further in addressing this limitation. Despite this observation, the bias information reported here provides a correction factor for comparison of vPCR quantification and spiking concentration of *E. coli* in commercial blood. 

Consistent with absolute quantification, calculations for percent viable cells also indicated an overestimate of live cells compared to spiked concentrations. As indicated above, this overestimate could be due to incomplete removal of target DNA. A more critical evaluation of how PMA treatment impacts amplification efficiency in vPCR needs to be conducted and taken into consideration during future development of vPCR for clinical use.

To summarize, the data reported here is the first to use vPCR to differentiate live and dead *E. coli* in whole blood. Herein we optimized the protocol by adding eukaryotic specific lysis prior to PMA exposure. The analytical parameters of the optimized protocol were within expected ranges. This optimized protocol can detect and quantify viable *E. coli* in blood in the presence of heat-killed cells. However, quantification from vPCR is higher than spiked concentration determined from plate count method. Ultimately, the data presented here provide the groundwork for further development of vPCR to detect and quantify live bacteria in blood that could facilitate early treatment of bacteremic infections.

## Data Availability

The data presented here are available on request.

## Supplementary Materials

The following supporting information can be downloaded at: https://www.mdpi.com/article/10.3390/microorganisms12040765/s1.
