# Supplementary material for: Optimization and Testing of a Commercial Viability PCR Protocol to Detect Escherichia coli in Whole Blood
Source: Microorganisms. 2024 Apr 10;12(4):765. doi: 10.3390/microorganisms12040765 (PMC11052410; doi:10.3390/microorganisms12040765)
Supplement: Supplementary file 1 [file microorganisms-12-00765-s001.zip › microorganisms-2918995-supplementary.pdf]

Supplemental Table S1: Average Ct values from 5 biological replicates from which linear regression was calculated. "HK" represents heat killed and number in sample column is CFU/mL (10^x CFU/mL).

Sample	Ct	Sample	Ct
10^8 Live + 0 HK	12.7	10^8 Live + 10^7 HK	10.9
10^7 Live + 0 HK	15.5	10^7 Live + 10^7 HK	13.7
10^6 Live + 0 HK	18.2	10^6 Live + 10^7 HK	16.1
10^5 Live + 0 HK	21.0	10^5 Live + 10^7 HK	19.2
10^4 Live + 0 HK	23.6	10^4 Live + 10^7 HK	22.4
10^3 Live + 0 HK	26.4	10^3 Live + 10^7 HK	24.8
10^2 Live + 0 HK	28.1		

Supplemental Table S2: Summary of intra- and inter-assay variability for copy number and Cq values. Variability is represented as percent coefficient of variation (CV%) or standard deviation (SD). "HK" represents heat killed and number in sample column is CFU/mL (10^x CFU/mL).

Sample	Copy# CV% intra	Copy# CV% inter	Cq SD intra	Cq SD inter
10^8 Live + 0 HK	5.7	4.6	0.06	1.64
10^7 Live + 0 HK	3.6	4.7	0.01	1.63
10^6 Live + 0 HK	6.8	6.0	0.05	1.84
10^5 Live + 0 HK	7.9	6.0	0.18	1.72
10^4 Live + 0 HK	7.4	4.9	0.10	1.25
10^3 Live + 0 HK	8.8	7.4	0.04	2.03
10^2 Live + 0 HK	10.0	10.1	0.23	2.44
10^8 Live + 10^7 HK	3.1	1.3	0.06	0.47
10^7 Live + 10^7 HK	7.2	3.9	0.17	1.37
10^6 Live + 10^7 HK	6.6	1.5	0.10	0.48
10^5 Live + 10^7 HK	5.9	2.0	0.09	0.60
10^4 Live + 10^7 HK	12.7	3.6	0.12	0.94
10^3 Live + 10^7 HK	15.4	6.9	0.17	1.71
10^2 Live + 10^7 HK	16.2	9.5	0.24	2.20
